# CRISPR/CasRx-Mediated RNA Knockdown Reveals That ACE2 Is Involved in the Regulation of Oligodendroglial Cell Morphological Differentiation

**DOI:** 10.3390/ncrna8030042

**Published:** 2022-06-06

**Authors:** Yukino Kato, Kenji Tago, Shoya Fukatsu, Miyu Okabe, Remina Shirai, Hiroaki Oizumi, Katsuya Ohbuchi, Masahiro Yamamoto, Kazushige Mizoguchi, Yuki Miyamoto, Junji Yamauchi

**Affiliations:** 1 Laboratory of Molecular Neurology, Tokyo University of Pharmacy and Life Sciences, Tokyo 192-0392, Japan; s179026@toyaku.ac.jp (Y.K.); s189072@toyaku.ac.jp (S.F.); s189014@toyaku.ac.jp (M.O.); rshirai@toyaku.ac.jp (R.S.); miyamoto-y@ncchd.go.jp (Y.M.); 2 Department of Biochemistry, Jichi Medical University, Tochigi 321-0498, Japan; ktago@jichi.ac.jp; 3 Tsumura Research Laboratories, Tsumura & Co., Ibaraki 200-1192, Japan; ooizumi_hiroaki@mail.tsumura.co.jp (H.O.); oobuchi_katsuya@mail.tsumura.co.jp (K.O.); hirokoma@h.email.ne.jp (M.Y.); mizoguchi_kazushige@mail.tsumura.co.jp (K.M.); 4 Department of Pharmacology, National Research Institute for Child Health and Development, Tokyo 157-8535, Japan

**Keywords:** oligodendrocyte, oligodendroglial cell, differentiation, ACE2, Ras

## Abstract

Angiotensin-converting enzyme 2 (ACE2) plays a role in catalyzing angiotensin II conversion to angiotensin (1–7), which often counteracts the renin-angiotensin system. ACE2 is expressed not only in the cells of peripheral tissues such as the heart and kidney, but also in those of the central nervous system (CNS). Additionally, ACE2 acts as the receptor required for the entry of severe acute respiratory syndrome coronavirus 2 (SARS-CoV-2), whose binding leads to endocytotic recycling and possible degradation of the ACE2 proteins themselves. One of the target cells for SARS-CoV-2 in the CNS is oligodendrocytes (oligodendroglial cells), which wrap neuronal axons with their differentiated plasma membranes called myelin membranes. Here, for the first time, we describe the role of ACE2 in FBD-102b cells, which are used as the differentiation models of oligodendroglial cells. Unexpectedly, RNA knockdown of ACE2 with CasRx-mediated gRNA or the cognate siRNA promoted oligodendroglial cell morphological differentiation with increased expression or phosphorylation levels of differentiation and/or myelin marker proteins, suggesting the negative role of ACE2 in morphological differentiation. Notably, ACE2′s intracellular region preferentially interacted with the active GTP-bound form of Ras. Thus, knockdown of ACE2 relatively increased GTP-bound Ras in an affinity-precipitation assay. Indeed, inhibition of Ras resulted in decreasing both morphological differentiation and expression or phosphorylation levels of marker proteins, confirming the positive role of Ras in differentiation. These results indicate the role of ACE2 itself as a negative regulator of oligodendroglial cell morphological differentiation, newly adding ACE2 to the list of regulators of oligodendroglial morphogenesis as well as of Ras-binding proteins. These findings might help us to understand why SARS-CoV-2 causes pathological effects in the CNS.

## 1. Introduction

Oligodendrocytes (also called oligodendroglial cells), which are generated from oligodendrocyte progenitors, are myelin-forming glial cells of the central nervous system (CNS) [1,2,3,4]. The major role of oligodendroglial cells is to produce myelin sheaths, membranes that extend from the oligodendroglial cells themselves. Myelin sheaths wrap around the neuronal axons. They help speed up the electric conduction velocity and physically protect axons [1,2,3,4]. Despite the well-known physiological roles of myelin sheaths, it remains unclear how oligodendroglial cells undergo morphological changes to differentiate before axonal myelination. Further detailed studies are urgently needed to understand the signaling molecular interaction underlying oligodendrocyte differentiation and/or myelination.

The renin-angiotensin system plays a central role in the regulation of blood pressure [5,6,7,8]. The angiotensin-converting enzyme homolog protease (ACE2) displays wide expression profiles compared to ACE1. ACE2 converts angiotensin II—a well-studied peptide in the renin-angiotensin system—to angiotensin [9,10], which specifically binds to a G protein-coupled receptor (GPCR) called Mas receptor (MAS). MAS is expressed in cell types such as smooth muscle cells, resulting in a decrease in blood pressure [5,6,7,8,9,10]. ACE2 is also well established as one of the major receptors required for the invasion of severe acute respiratory syndrome coronavirus 2 (SARS-CoV-2) into cells [11,12,13,14]. It is thus thought that ACE2-expressing cells are found in many tissues, including nerve ones, and are severely damaged in the early onset of the disease [11,12,13,14].

Herein, we identify ACE2 itself as the negative regulator of cell morphological differentiation using FBD-102b cells, which are used as model cell lines undergoing differentiation of oligodendroglial cells [15,16]. RNA knockdown of ACE2 using the clustered regularly interspaced short palindromic repeats (CRISPR)-Cas system (CasRx-mediated guide RNA [gRNA]) [17,18] or its small interfering RNA (siRNA) resulted in promoting morphological differentiation. The intracellular region of ACE2 resulted in interacting with the active GTP-bound forms of Ras, which is one of the small GTPase family members that are involved in the regulation of intracellular signaling molecules leading to oligodendroglial cell differentiation and, in turn, myelination [19,20,21,22]. Therefore, as expected, inhibition of Ras inhibited their differentiation. These results reveal the presence of the ACE2 intracellular region and Ras signaling module underlying oligodendroglial cell morphological differentiation, possibly explaining why SARS-CoV-2 infection causes pathological effects on the CNS through ACE2.

## 2. Materials and Methods

### 2.1. Cell Culture and Differentiation

Mouse brain oligodendrocyte precursor cell line FBD-102b (kindly provided by Dr. Y. Tomo-oka (Tokyo University of Science, Chiba, Japan and Riken, Saitama, Japan)) was cultured on standard 3.5 to 10 cm cell and tissue culture dishes (Nunc Cell Culture Plastics, Thermo Fisher Scientific, Waltham, MA, USA) in DMEM/F12 mixed medium (Nacalai Tesque, Kyoto, Japan) containing 10% heat-inactivated fetal bovine serum (FBS) and antibiotics containing penicillin and streptomycin in 5% CO_2_ at 37 °C.

To induce differentiation, FBD-102b cells were cultured on tissue culture dishes coated with 100 μg/mL of polylysine in DMEM/F12 without FBS for 72 h in 5% CO_2_ at 37 °C [15,16]. Cells bearing multiple processes (primary and secondary branches) from the cell bodies were identified as differentiated [15,16]. Differentiation was assessed in terms of three levels of completeness: Category 1 included cells with fewer than two primary branches; Category 2 included cells with fewer than three primary branches and without secondary branches (additional branches from the primary branches); and Category 3 included cells with more than three primary branches and with secondary branches as well as with widespread membranes. Category 1 was considered to be the phenotypes before differentiation, whereas Category 3 was to be the differentiated phenotypes. Category 2 corresponded to these intermediate phenotypes.

### 2.2. Isolation, Culture, and Differentiation of Rat Oligodendrocyte Precursor Cells

Oligodendrocyte precursor cells were isolated from embryonic day 15 Sprague–Dawley rats [15,16]. Briefly, cerebral cortices were dissected, dissociated with 0.25% trypsin, triturated, and passed through mesh with 70 μm pores. Cells were collected, resuspended in MEM (Thermo Fisher Scientific, Waltham, MA, USA) containing 10% FBS and antibiotics containing penicillin and streptomycin, and seeded on poly-L-lysine-coated cell and tissue culture dishes. After two passages, cells were cultured on non-coated Petri dishes. The medium was changed to DMEM (Thermo Fisher Scientific, Waltham, MA, USA)-based serum-free growth medium containing 10 ng/mL platelet-derived growth factor (PDGF)-AA and 10 ng/mL basic fibroblast growth factor (bFGF) and N2 supplement solution (Thermo Scientific) on the second day of culture [15,16]. The cells were cultured for an additional 2 days and used as 97.5%-purified, type A PDGF receptor (PDGFRA)-positive oligodendrocyte precursor cells (a proliferating state before differentiation) [15,16].

To induce differentiation of oligodendrocyte precursor cells, cells were cultured with a differentiation medium containing 20 ng/mL triodothyronine and 20 ng/mL thyroxine without growth factors. After 3 days, cells were allowed to differentiate into mature oligodendrocytes bearing multiple processes [15,16].

### 2.3. Nucleotide Transfection for siRNA, gRNA, or Oligopeptide Expression Plasmid

Cells were transfected with the respective synthesized 21-mer siRNAs with dTdT (Fasmac, Kanagawa, Japan) using the ScreenFect siRNA transfection kit (FUJIFILM, Tokyo, Japan), according to the manufacturer’s instructions. The medium was replaced 4 h after transfection and cells were generally used for experiments 48 h after transfection.

Cells were also transfected with the respective plasmids (pEF1a (pEF-BOS modified type) encoding CasRx and pSINmU6 encoding gRNA or pcDNA3.1-N-EGFP-human ACE2 intracellular domain) using the ScreenFect A transfection kit (FUJIFILM, Tokyo, Japan), according to the manufacturer’s instructions. The medium was replaced 4 h after transfection and cells were generally used for experiments 48 h after transfection. All key reagents and antibodies used are listed in Table 1.

### 2.4. Reverse Transcription-Polymerase Chain Reaction (RT-PCR)

The cDNAs were prepared from Isogen (Nippon Gene, Tokyo, Japan)-extracted total RNA with a PrimeScript RT Master Mix kit (Takara Bio, Shiga, Japan) in accordance with the manufacturer’s instructions.

PCR amplification from RT products was performed using Gflex DNA polymerase (Takara Bio) with 36 to 40 cycles, each consisting of a denaturation reaction at 98 °C (0.2 min), an annealing reaction at 65.5 °C (0.25 min), and an extension reaction at 68 °C (0.5 min). The resultant PCR products were applied to 1% to 2% agarose gels.

### 2.5. Immunoblotting and Immunoprecipitation

Cells were lysed in lysis buffer (50 mM HEPES-NaOH, pH 7.5, 150 mM NaCl, 20 mM MgCl_2_, 1 mM dithiothreitol, 1 mM phenylmethane sulfonylfluoride, 1 μg/mL leupeptin, 1 mM EDTA, 1 mM Na_3_VO4, 10 mM NaF, and 0.5% NP-40). Their supernatants were quantified by centrifugation using a Coomassie Brilliant Blue Protein Assay Kit (Nacalai Tesque, Kyoto, Japan) and denatured in denaturing sample buffer (Nacalai Tesque, Kyoto, Japan). The samples (20 μg/sample) were equally separated on 10 to 15% sodium dodecylsulfate–polyacrylamide gels (Nacalai Tesque, Kyoto, Japan). The electrophoretically separated proteins were transferred to PVDF membranes, blocked with skim milk, and immunoblotted using primary antibodies, followed by peroxidase-conjugated secondary antibodies. The bound antibodies were detected by X-ray film exposure using the chemiluminescence method. The images on the films were converted to TIFF image files using a Canon LiDE 400 scanner (Canon, Tokyo, Japan) and its driver software (Canon, Tokyo, Japan). The band pixels were measured using Image J software (Bethesda, MD, USA). It was calculated as a relative pixel value when the control experiments were set to 100%. Each image in the figures is representative of three independent experimental results.

The supernatant of cell lysates transfected was collected for immunoprecipitation in the presence or absence of 1 μM of GTP or GDP. The supernatants were mixed with protein G resin that had been absorbed with an antibody [15,16]. Immunoprecipitates from the supernatant of the cell lysates were denatured, subjected to sodium dodecylsulfate–polyacrylamide gels, blotted onto PVDF membranes, and immunoblotted with an anti-Ras antibody.

### 2.6. Affinity-Precipitation Assay for Ras

The supernatants of cytoplasmic lysates of transfected cells were collected. The supernatants were mixed with glutathion resin that had been absorbed with recombinant glutathion-S-transferase(GST)-tagged Ras-binding domain proteins to monitor free, active GTP-bound Ras [22]. Affinity-precipitates from the supernatant of the cell lysates were denatured, subjected to sodium dodecylsulfate–polyacrylamide gels, blotted onto PVDF membranes, and immunoblotted with the respective antibodies.

### 2.7. Statistical Analyses

Values are means ± standard deviation (SD) from separate experiments. Intergroup comparisons were made using the unpaired *t*-test with Student’s or Welch’s correction using Microsoft Excel (ver. 2019, Microsoft, Redmond, WA, USA). For multiple comparisons, statistical analyses were performed according to the chi-square goodness-of-fit test using Microsoft Excel.

### 2.8. Ethics Statement

Gene recombination techniques in cells were performed in accordance with protocols approved by the Tokyo University of Pharmacy and Life Sciences Gene and Animal Care Committees (LS28-20 and LSR3-011).

## 3. Results

### 3.1. ACE2 Negatively Regulates Oligodendroglial Cell Morphological Differentiation

**Figure 1:**
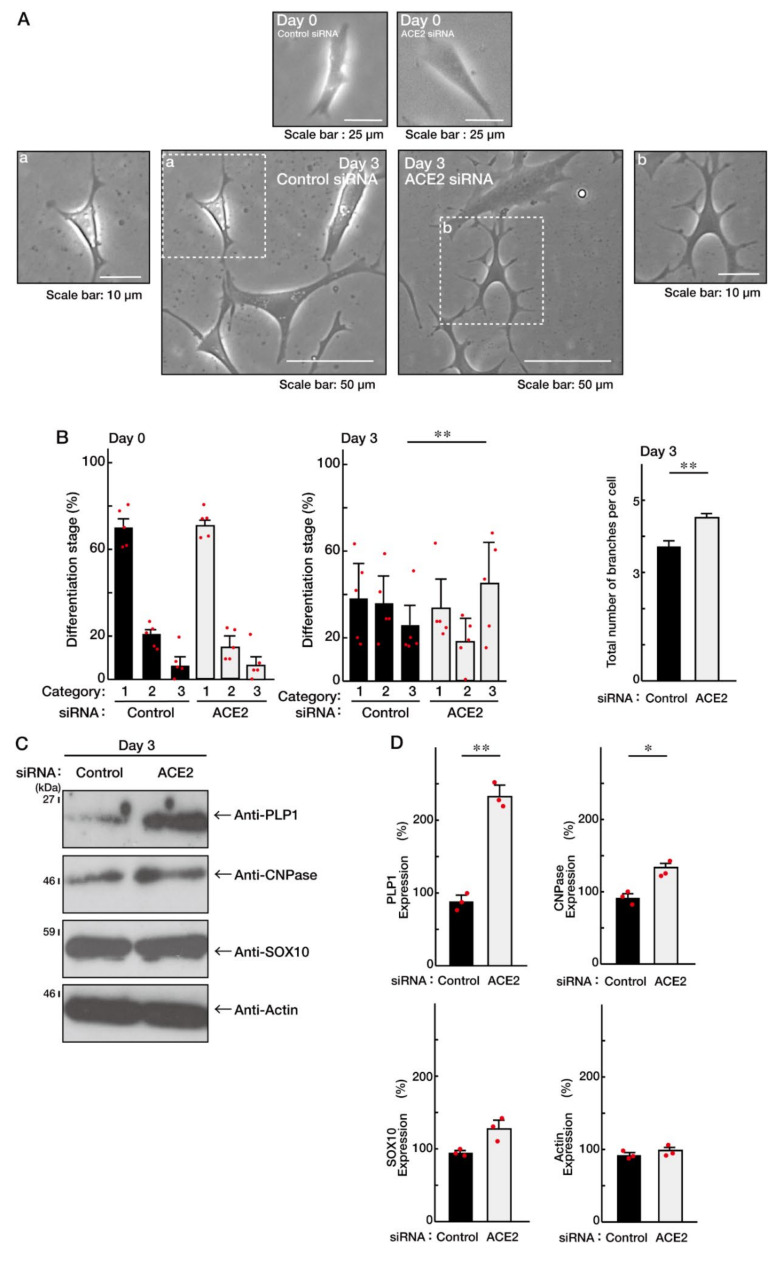
Knockdown of ACE2 by siRNA promotes oligodendroglial cell morphological differentiation. (**A**,**B**) FBD-102b cells were transfected with control or ACE2 siRNA and were allowed to be differentiated for 0 or 3 days. Differentiation efficiencies were divided into three categories and depicted in graphs (**, *p* < 0.01; *n* = 5 fields). Category 1 included cells with fewer than two primary branches; Category 2 included cells with fewer than three primary branches and without secondary branches; and Category 3 included cells with more than three primary branches and with secondary branches as well as with widespread membranes. Category 1 was considered to be the phenotypes before differentiation, whereas Category 3 was considered to be the differentiated phenotypes. Category 2 corresponded to these intermediate phenotypes. The number of branches in cells is also counted and shown (**, *p* < 0.01; *n* = 50 cells). (**C**,**D**) Cells at 3 days following the induction of differentiation were collected, lysed, and immunoblotted with an antibody against PLP1, CNPase, SOX10, or actin. Immunoreactive band intensities were also compared to be depicted in graphs (**, *p* < 0.01 and *, *p* < 0.05; *n* = 3 blots).

**Figure 2:**
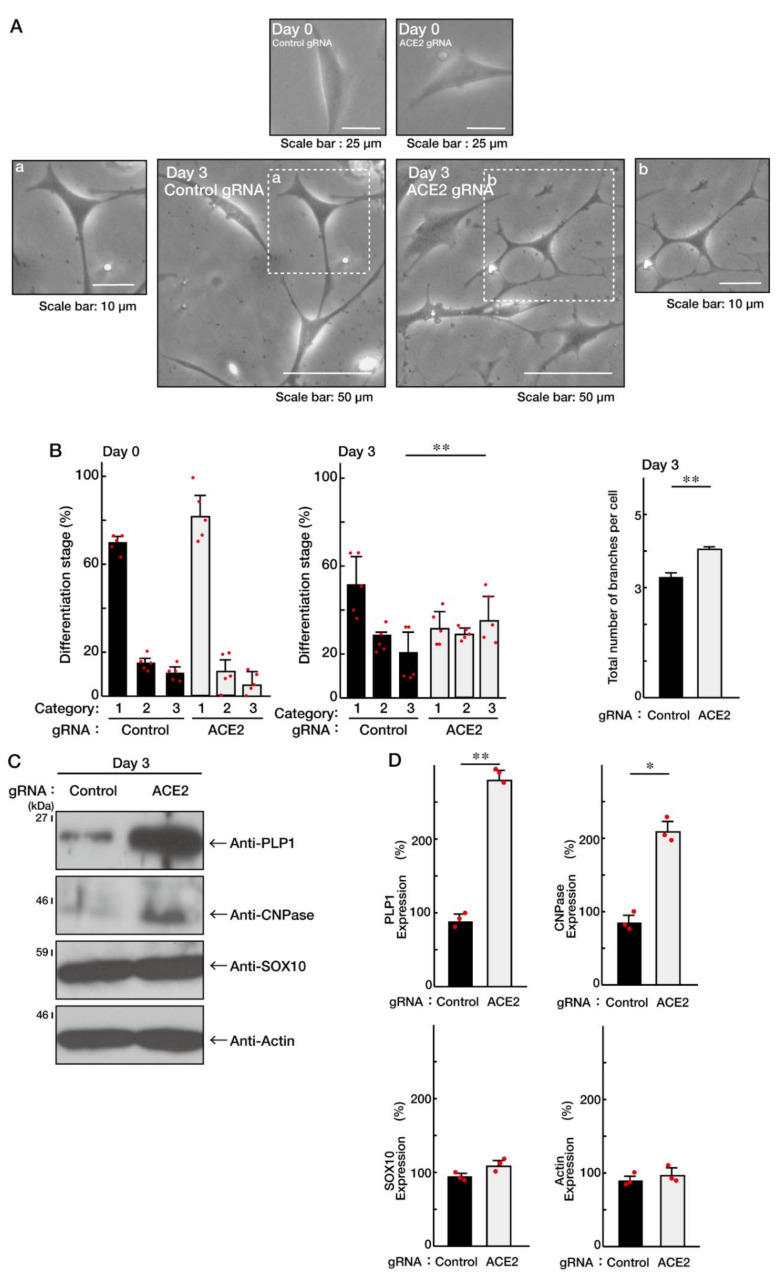
Knockdown of ACE2 by gRNA promotes oligodendroglial cell morphological differentiation. (**A**,**B**) FBD-102b cells were transfected with the plasmids encoding ACE2 gRNA with CasRx or control plasmids and were allowed to be differentiated for 0 or 3 days. Differentiation efficiencies were divided into three categories and depicted in graphs (**, *p* < 0.01; *n* = 5 fields). The number of branches in cells is also counted and shown (**, *p* < 0.01; 50 cells). (**C**,**D**) Cells at 3 days following the induction of differentiation were collected, lysed, and immunoblotted with an antibody against PLP1, CNPase, SOX10, or actin. Band intensities were also compared to be depicted in graphs (**, *p* < 0.01 and *, *p* < 0.05; *n* = 3 blots).

To investigate whether ACE2 is involved in the regulation of oligodendroglial cell morphological differentiation, we specifically knocked down ACE2 using the cognate siRNAs in FBD-102b cells. The knockdown resulted in promoting morphological differentiation with multiple processes from cell bodies (Figure 1A,B; Figure S1A). These results were consistent with the increased expression of differentiation and myelin markers proteolipid protein 1 (PLP1) and cyclic nucleotide 3′-phosphodiesterase (CNPase; Figure 1C,D), indicating that ACE2 is a negative regulator of oligodendroglial cell morphological differentiation. In contrast, oligodendrocyte-lineage cell marker SRY-related HMG-box protein 10 (SOX10) and control actin proteins were comparable in cells knocked down with control or ACE2 siRNA. Similarly, to confirm that ACE2 is involved in promoting morphological differentiation, we specifically knocked down ACE2 using CasRx-mediated gRNA [17,18]. The knockdown revealed that ACE2 negatively regulates morphological differentiation (Figure 2A,B; Figure S1B), consistent with the results that the expression levels of PLP1 and CNPase were upregulated following its knockdown (Figure 2C,D). In contrast, the expression levels of SOX10 and actin proteins were comparable.

To confirm the correlation between primary cultured cells and FBD-102b cells, primary oligodendrocyte progenitor cells were isolated from rat brains. Cells were allowed to be differentiated. Inhibition of ACE2 with MLN-4760, an ACE2 inhibitor [23] had a tendency to promote differentiation with increased marker protein expression in primary cultured cells (Figure S2), consistent with the results from FBD-102b cells (Figure S3).

**Figure 3:**
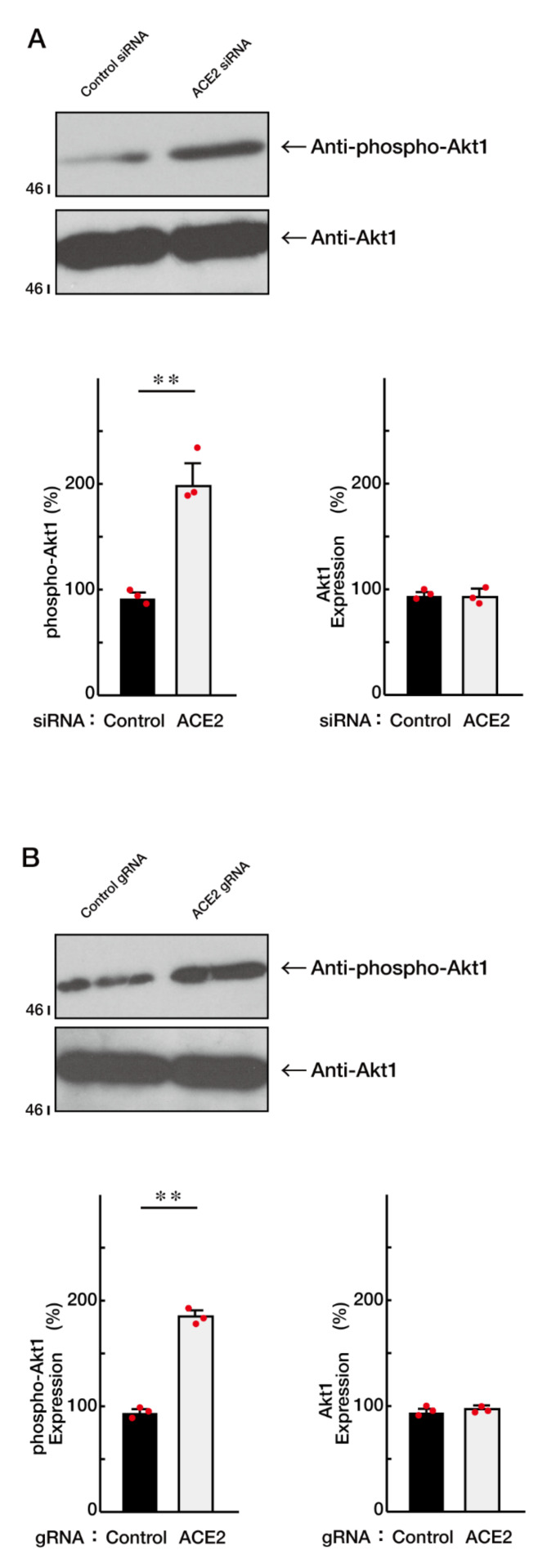
Knockdown of ACE2 promotes Akt1 phosphorylation whose site is essential for Akt1 activation. (**A**) The lysates of siRNA-knocked down FBD-102b cells were used for immunoblotting with an anti-phosphorylated (phospho) Akt1 or Akt1. These two different blots were performed from the same samples. Band intensities were also compared to be depicted in graphs (**, *p* < 0.01; *n* = 3 blots). (**B**) The lysates of gRNA-knocked down cells were used for immunoblotting with an anti-phosphorylated (phospho) Akt1 or Akt1. These two different blots were performed from the same samples. Band intensities were also compared to be depicted in graphs (**, *p* < 0.01; *n* = 3 blots).

It is well known that the activities of Akt kinase are critically required for oligodendroglial cell differentiation and, in turn, myelination as well as remyelination [24,25]. Knockdown of ACE2 with the cognate siRNA increased the levels of Akt phosphorylation, whose position at Ser-473 is essential for Akt activation (Figure 3A). Similar results were obtained in the case of the knockdown using CasRx-mediated gRNA (Figure 3B), confirming that ACE2 itself is a negative regulator of morphological differentiation.

### 3.2. ACE2 Preferentially Interacts with an Active GTP-Bound Ras to Interact with Ras

**Figure 4:**
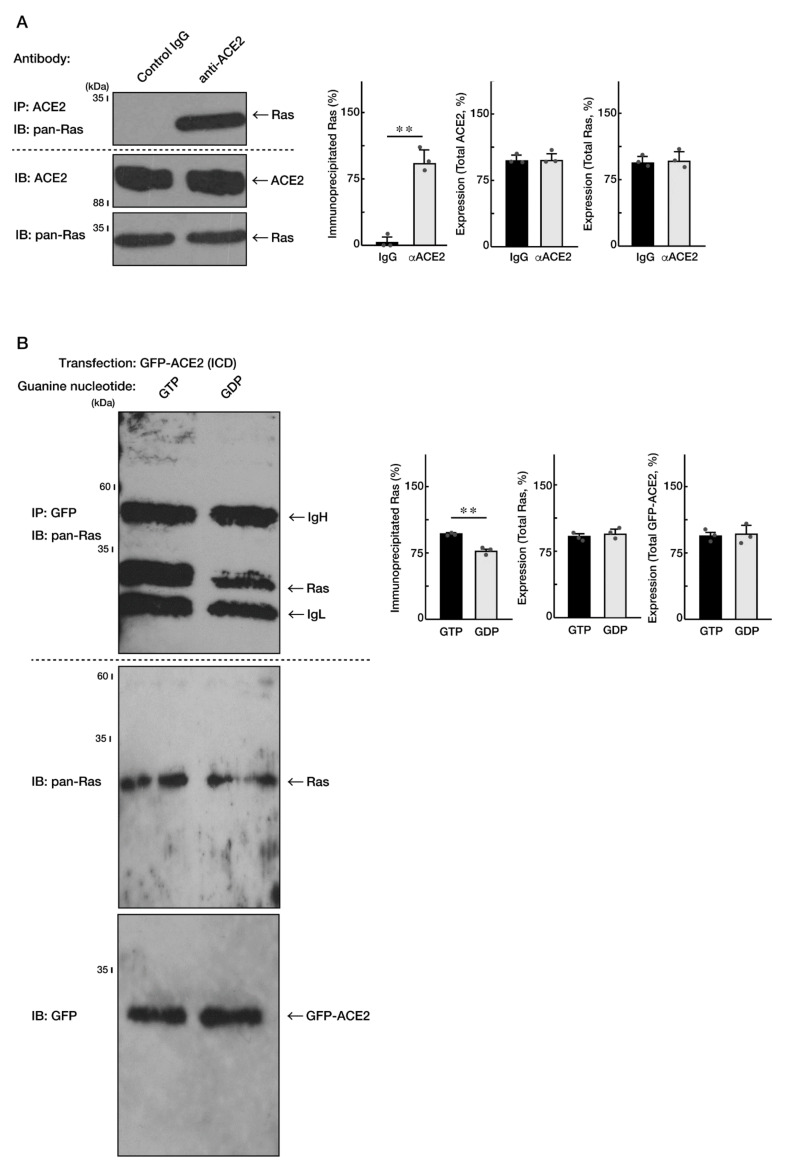
The intracellular domains of ACE2 interacts with Ras. (**A**) The lysates of FBD-102b cells were immunoprecipitated with an anti-ACE2 IgG or control IgG and then immunoblotted with an anti-Ras (pan-Ras) antibody. Total ACE2 and Ras proteins were also shown to confirm whether their expression levels are comparable. Band intensities were also compared to be depicted in graphs (**, *p* < 0.01; *n* = 3 blots). (**B**) The lysates of cells transfected with the plasmid encoding GFP-tagged ACE2 intracellular domain (ICD) were immunoprecipitated with an anti-GFP antibody in the presence of 1 μM of GTP or GDP and then immunoblotted with an anti-Ras antibody. Total Ras and GFP-tagged proteins were also shown to confirm whether their expression levels are comparable. IgH and IgL indicate the probable heavy and light chains of immune globulins for immunoprecipitation experiments, respectively. Band intensities were also compared to be depicted in graphs (**, *p* < 0.01; *n* = 3 blots).

The BioGRID database (see the website, https://thebiogrid.org/, accessed on 1 May 2022) shows that Ras is the potential binding partner of ACE2. It is also well known that Ras plays a key role in oligodendroglial cell morphological differentiation and, in turn, myelination [19,20,21]. Therefore, we investigated their interaction using an immunoprecipitation technique in FBD-102b cells. Endogenous ACE2 was immunoprecipitated with an anti-ACE2 antibody and then immunoblotted with an anti-Ras antibody. An immunoreactive band for Ras was specifically observed in the presence of an anti-ACE2 antibody but not its absence (Figure 4A). In these experiments, the expression levels of ACE2 and Ras were comparable in both conditions.

To explore whether the ACE2-Ras interaction is dependent on each form of guanine-nucleotides, we transfected the plasmids encoding the intracellular domain of ACE2 into cells and performed co-immunoprecipitation studies with Ras. The intracellular domain of ACE2 preferentially formed an immune complex with the GTP-bound, endogenous Ras, which can become formed in the presence of excessive GTP (Figure 4B). Similar results were obtained in the case of the interaction with endogenous Ras downstream effectors phosphoinositide 3-kinase α and β (Figure S4; Figure S5), which are known to be direct upstream regulators of Akt kinase [19,20,21,24,25,26,27]. Taken together with the above results, it is suggested that Ras and the Ras signaling complex can be trapped to ACE2.

**Figure 5:**
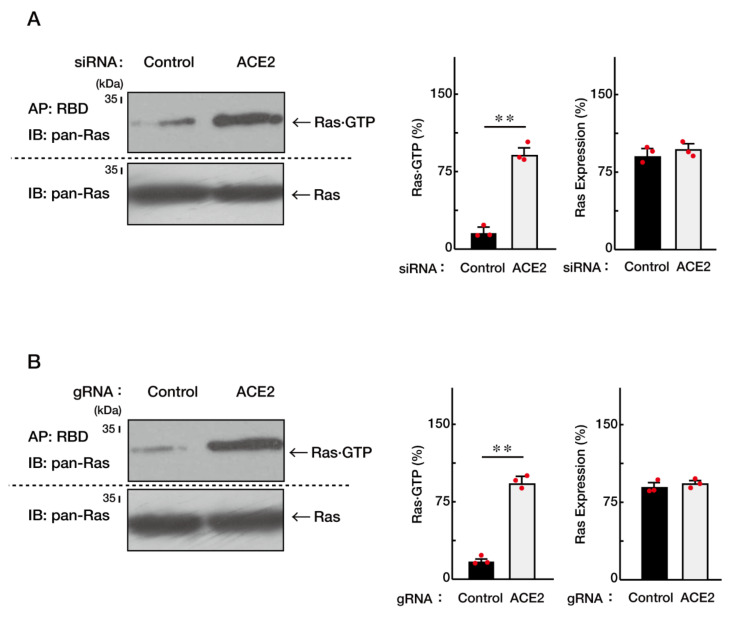
Knockdown of ACE2 promotes the GTP-bound form of Ras in affinity-precipitation assay. (**A**) The lysates of siRNA-knocked down FBD-102b cells were used for affinity-precipitation (AP) assay, monitoring activated GTP-bound Ras with Ras-binding domain (RBD). Total Ras proteins were also shown. Band intensities were also compared to be depicted in graphs (**, *p* < 0.01; *n* = 3 blots). (**B**) The lysates of gRNA-knocked down cells were used for affinity-precipitation assay to monitor active GTP-bound Ras. Total Ras proteins were also shown. Band intensities were also compared to be depicted in graphs (**, *p* < 0.01; *n* = 3 blots).

Therefore, we checked whether ACE2 knockdown increases the GTP-bound form of Ras. Either knockdown of ACE2 using siRNA or CasRx-mediated gRNA indeed increased the GTP-bound form of Ras (Figure 5A,B). In contrast, their knockdown did not change the total amounts of Ras.

### 3.3. Ras Positively Regulates Morphological Differentiation

**Figure 6:**
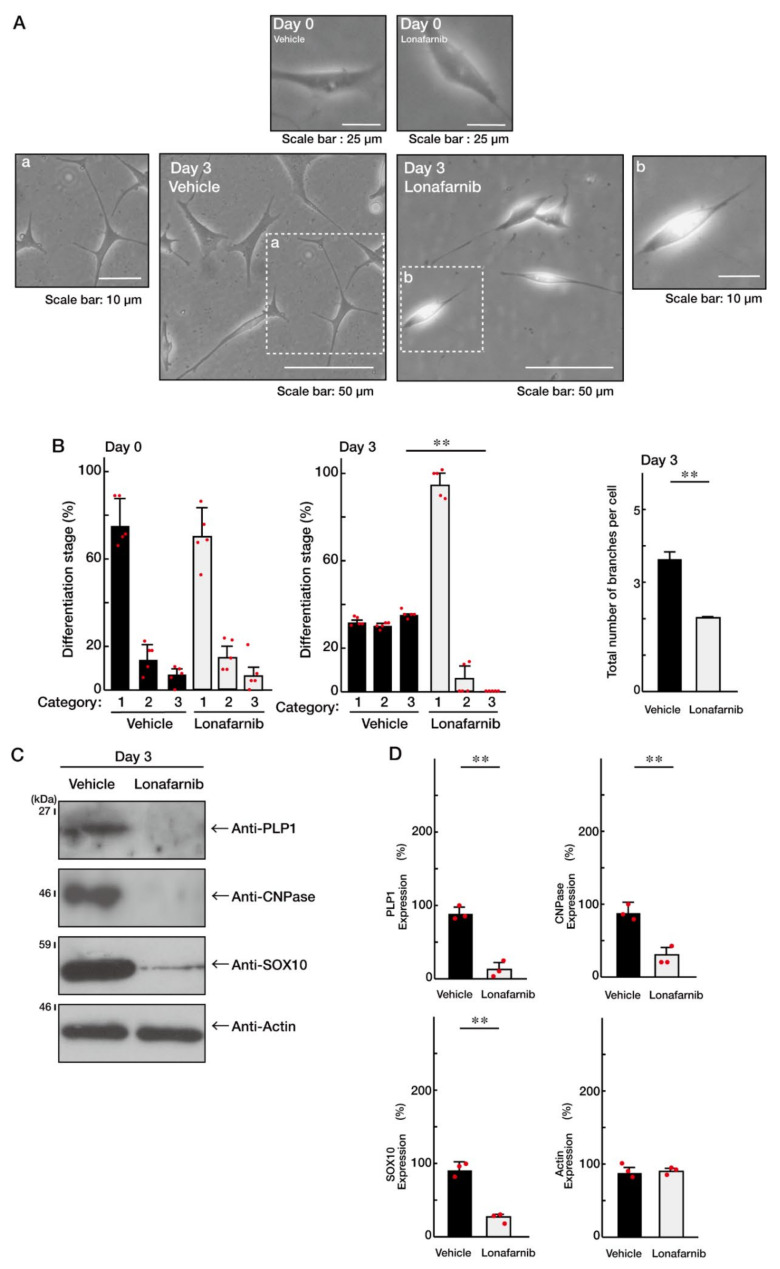
Ras inhibition inhibits oligodendroglial cell morphological differentiation. (**A**,**B**) FBD-102b cells were treated with vehicle or Lanafamib (5 μM) and were allowed to be differentiated for 0 or 3 days. Differentiation efficiencies were divided into three categories and depicted in graphs (**, *p* < 0.01; *n* = 5 fields). The number of branches in cells is also counted and shown (**, *p* < 0.01; 50 cells). (**C**,**D**) Cells at 3 days following the induction of differentiation were collected, lysed, and immunoblotted with an antibody against PLP1, CNPase, SOX10, or actin. Immunoreactive band intensities were also compared to be depicted in graphs (**, *p* < 0.01; *n* = 3 blots).

**Figure 7:**
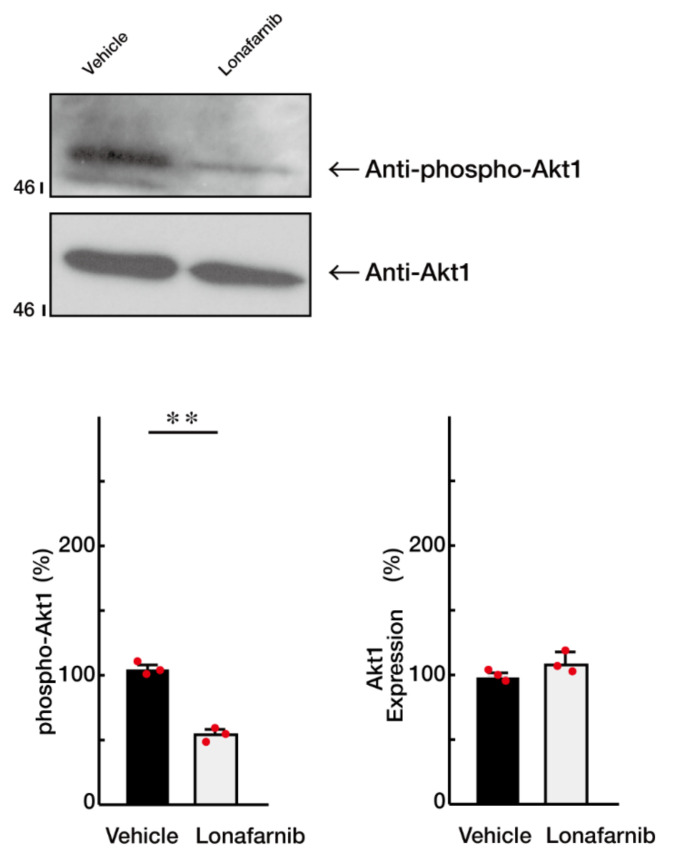
Ras inhibition decreases Akt1 phosphorylation. The lysates of vehicle or Lanafamib-treated FBD-102b cells were used for immunoblotting with an anti-phosphorylated (phospho) Akt1 or Akt1. These two different blots were performed from the same samples. Band intensities were also compared to be depicted in graphs (**, *p* < 0.01; *n* = 3 blots).

Finally, we sought to confirm that Ras is the positive regulator of oligodendroglial cell morphological differentiation in our experimental conditions. We incubated cells with Lonafarnib, a specific inhibitor of Ras [28], and found that Lonafarnib greatly inhibited oligodendroglial cell morphological differentiation in FBD-102b cells (Figure 6A,B). The expression levels of SOX10—as well as those of PLP1 and CNPase—were downregulated following the treatment with Lonafarnib (Figure 6C,D), suggesting that Ras is critically involved in the promotion of morphological differentiation. In contrast, the expression levels of actin were comparable. In addition, the treatment of cells with Lonafarnib greatly decreased the levels of Akt phosphorylation (Figure 7). These results are consistent with the fact that Ras is a positive regulator of oligodendroglial cell differentiation and myelination [19,20,21,24,25,26].

**Figure 8:**
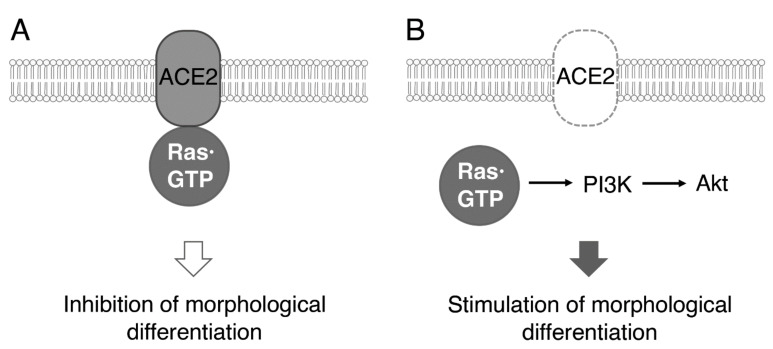
Schematic diagram of the relationship of the ACE2 and Ras signaling with morphological differentiation in FBD-102b cells. ACE2, acting through capturing Ras·GTP, inhibits oligodendroglial cell morphological differentiation (**A**), whereas ACE2 knockdown stimulates morphological differentiation through a possible pathway linking to Akt kinase (**B**).

Collectively, since ACE2 as the transmembrane molecule results in interacting preferentially with the active, GTP-bound form of Ras, it is possible that in the presence of ACE2, ACE2 capture the active Ras, inhibiting downstream cytoplasmic molecules including Akt kinase signaling molecules (see the schematic diagram in the Figure 8). Their sequential activation is essential for triggering oligodendroglial cell differentiation and myelination [19,20,21,24,25,26]. On the other hand, in the absence or low amounts of ACE2, GTP-bound Ras can stimulate downstream cytoplasmic signaling to promote differentiation.

## 4. Discussion

ACE2 is one of the major receptors needed for the entry of SARS-CoV-2 into cells. The cells, which can be infected with SARS-CoV-2, distribute not only in the cells of respiratory organs but also in those of the nervous system. One target cell of SARS-CoV-2 in the CNS is the oligodendroglial cell [11,12,13,14]. It remains unclear how SARS-CoV-2 infection through ACE2 causes the pathological effects in the CNS. When oligodendroglial cells encounter physical and physiological damage in diseases and disease models, often caused experimentally by means of using genetic modification technologies, it is suggested that they exhibit abnormal cell differentiation phenotypes [3]. For example, their differentiation can become severely inhibited [3,4] or excessively promoted [29,30]. As a result, the cell composition in the CNS collapses and neuronal cells can also be seriously damaged [11,13,14]. In this study, first, we knocked down ACE2 using the CRISPR-Cas system (CasRx-mediated gRNA) or siRNA and identified ACE2 itself as the negative regulator of oligodendroglial cell morphological differentiation. In addition, the intracellular region of ACE2 preferentially interacted with the active GTP-bound form of small GTPase Ras. Then, inhibition of Ras with a Ras inhibitor resulted in inhibiting their morphological differentiation, consistent with the fact that Ras signaling is required for differentiation and, in turn, myelination in oligodendrocyte cells [19,20,21,24,25,26]. It is suggested that, in the presence of ACE2, it inhibits morphological differentiation by trapping Ras. In contrast, in the absence or with low amounts of ACE2, Ras can be released from its intracellular region to mediate morphological differentiation. This is the first report on the role of ACE2 in oligodendroglial cell morphological differentiation. These results might explain how SARS-CoV-2 causes the pathological effects through ACE2. From these results, we speculate that the functional loss of ACE2 by binding to SARS-CoV-2 likely leads to abnormal differentiational phenotypes in oligodendroglial cells of SARS-CoV-2-infected brains. One of the reasons for the various pathological phenotypes observed in SARS-CoV-2-infected brains [11,12,13,14] may be due to their abnormal differentiational phenotypes, since it is thought that proper interactions between oligodendroglial cells with normal morphologies and neuronal cells are needed for triggering suitable roles in neurons and neuronal tissues [1,2,3,4].

There have been some reported examples of transmembrane proteins trapping signaling molecules to inhibit positive cellular functions. In cultured chick ciliary ganglion neurons and mouse spinal and sensory neurons, the intracellular domain of p75 nerve growth factor receptor (p75^NTR^, also called low-affinity neurotrophin receptor) directly binds to RhoA, which belongs to Rho family small GTPases (differing from Ras family GTPases) to inhibit RhoA activities. The Rho GTPase inhibition caused by their interaction leads to axonal growth [31]. The p75^NTR^ intracellular domain also binds to Rho GDP dissociation inhibitor (Rho-GDI), which blocks RhoA’s activation following the upstream signals. Structural changes of the p75^NTR^ intracellular domain are involved in facilitating the release of RhoA from Rho-GDI [32]. In static states, p75^NTR^ itself blocks the RhoA activities to inhibit the cellular functions. Another example is the case of Rnd1, a member of Rho family small GTPase. The intracellular domain of Plexin-B1, which is the receptor for a ligand molecule controlling axon guidance, binds to Rnd1. This interaction is a unique one and is required for stimulating the GTPase activities of R-Ras, decreasing cellular R-Ras activities to collapse the growth cone in rat hippocampal neurons [33]. This molecular interactive mechanism is conserved in other plexin families and other small GTPase families as well [34]. It is possible that ACE2 itself has GDI or GAP activities which affect oligodendroglial cell morphological differentiation; however, the ACE2 intracellular region is likely too short to have homologies with GDI or GAP for Ras [35]. Even if ACE2 had GDI or GAP activities, it is clear that ACE2 itself acts as the negative regulator of Ras signaling during oligodendroglial cell morphological differentiation.

It has been well established that the renin-angiotensin system, centered on angiotensin I, plays a central role in the regulation of blood pressure. Juxtaglomerular apparatus-derived renin protease converts angiotensinogen proteins such as preproproteins, which are secreted in blood from organs such as the liver, to angiotensin I. ACE (also called ACE1), which is generated by organs such as the lung, converts angiotensin I to angiotensin II in the blood. Angiotensin II is a ligand that mainly binds to angiotensin receptor 1 (AT1) as the cell surface GPCR, resulting in an increase in blood pressure [1,2,3,4,5,6,7,8]. In contrast, ACE2 converts angiotensin II to angiotensin (1–7), which specifically binds to the cell surface MAS, resulting in a decrease in blood pressure [9,10]. ACE2 displays wider expression profiles differing from ACE1. ACE2 has a larger variety of expression profiles and functions than initially expected. Additionally, ACE2 is expressed in neuronal bodies in the brain [11,12,13]. ACE2 might convert angiotensin II to angiotensin (1–7) in oligodendroglial cells as well as in neuronal cells in the brain. Angiotensin (1–7) activation of MAS might affect morphological differentiation in oligodendroglial cells. MAS is primarily coupled to Gq-type heterotrimeric GTP-binding proteins (Gq subfamily proteins) to trigger calcium signaling [9,10], which plays a key role in oligodendroglial cell differentiation [36,37,38]. It is possible that the subtle regulation of calcium signaling in oligodendroglial cells, acting through ACE2 and MAS, may affect their differentiation. Although the direct relationship between Ras as an ACE2 interactive partner and calcium signaling remains unknown, their potential relationship may be worth studying.

Herein, we identify ACE2 as the negative regulator of oligodendroglial cell morphological differentiation. We also find that morphological differentiation requires Ras as an ACE2 interactive partner. Regulating cell morphological differentiation in oligodendroglial cells involves two types of molecules: one is to promote oligodendroglial cell morphological differentiation and the other is to inhibit morphological differentiation. For example, neuregulin-1 and the receptor ErbB2/3 as well as VCAM1 are included in the former regulatory molecules [1,2,3,4,39] and, in contrast, ACE2 is included in the latter. It is thought that these two types of molecules act in concert to fine-tune morphogenesis. Further detailed studies will promote our understanding not only of the molecular mechanism by which ACE2—acting by capturing Ras—inhibits differentiation, but also of whether differentiation requires the enzymatic activities of ACE2, acting cooperatively with ACE1. Additional studies are needed to determine whether ACE2 is actually involved in the regulation of myelination following differentiation in oligodendroglial cells in mice as well as in primary cells. Studies along these lines may help us to clarify the relationship of ACE2 with SARS-CoV-2-associated myelin-related diseases [40]. These studies may lead to the development of specific therapeutic procedures and drug-target-specific medicines for SARS-CoV-2-associated myelin-related diseases [40] as well as for some of their pathological effects in the CNS [13,14].

## Figures and Tables

**Table 1:** Key antibodies and chemical sources. All key materials such as antibodies are shown.

Reagent or Source	Company or Source	Cat. No.	Lot. No.	Concentration Used
Antibodies				
Anti-proteolipid protein (PLP) 1	Atlas Antibodies	HPA004128	B115828	IB, 1/500
Anti-cyclic nucleotide 3′-phosphodiesterase (CNPase)	BioLegend	836404	B278794	IB, 1/500
Anti-SRY-related HMG-box protein (SOX) 10	Santa Cruz Biotechnology	sc-365692	F1621	IB, 1/500
Anti-Actin	MBL	M177-3	007	IB, 1/80,000
Anti-phospho-Akt1 (pSer473, which is essential for Akt1 activation)	Cell Signaling Technology	9018S	13	IB, 1/500
Anti-Akt1	Cell Signaling Technology	2938S	2	IB, 1/500
Anti-angiotensin-converting enzyme (ACE) 2	Santa Cruz Biotechnology	sc-390851	F2520	IB, 1/500
Anti-pan-Ras	Santa Cruz Biotechnology	sc-166691	H1220	IB, 1/500
Anti-green fluorescence protein (GFP)	Nacalai Tesque	04404-84	M7H8151	immunoprecipitation, 1 mg for 1 mg proteins
Anti-GFP	MBL	M048-3	066	IB, 1/1000
Anti-DDDDK	MBL	M185-3L	002	IB, 1/10,000
Anti-IgG (H+L chain) (Mouse) pAb-HRP	MBL	330	365	IB, 1/5000
Anti-IgG (H+L chain) (Rabbit) pAb-HRP	MBL	458	353	IB, 1/5000
Anti-PI 3-kinase p110α (PI3Ka)	Santa Cruz Biotechnology	sc-518070	D2519	IB, 1/250
Anti-PI 3-kinase p110β (PI3Kb)	Santa Cruz Biotechnology	sc-376641	H1320	IB, 1/250
Key chemicals				
guanosine triphosphate (GTP)	Merk	G8634-1MG	056K1555	1 μM for immunoprecipitation
guanosine diphosphate (GDP)	Merk	371545	B62411	1 μM for immunoprecipitation
Glutathion-sepharose 4B	GE Healthcare	17-0756-05	10058508	45 μL of 33% slurry (containing 30 μg of glutathion-S-transferase (GST)-Ras binding domain proteins) for 1 mg of total proteins in cell lysates
Protein G-sepharose 4FastFlow	GE Healthcare	17-0618-01	10081061	45 μL of 33% slurry for 1 mg of total proteins in cell lysates
Lonafarnib as a Ras inhibitor	CAYMAN CHEMICAL	11746	10081061	5 μM for cell treatment
MLN-4760 as an ACE2 inhibitor	MedChemExpress	HY-19414	305335-31-3	1 nM for cell treatment
Key reagents				
ScreenFect TM siRNA Transfection Reagent	FUJIFILM Wako Pure Chemical Corporation	292-75013	CAM0357	
ScreenFect TM Dilution Buffer	FUJIFILM Wako Pure Chemical Corporation	194-18181	SKF5794	
ImmunoStar Zeta	FUJIFILM Wako Pure Chemical Corporation	295-72404	LEP1844	
Chemi-Lumi One Ultra	Nacalai Tesque	11644-24	L1G6389	
Skim Milk Powder	FUJIFILM Wako Pure Chemical Corporation	190-12865	SKG4901	
Western blotting (WB) Stripping Solution	Nacalai Tesque	05364-55	L5M5218	
Gflex DNA Polymerase	TaKaRa Bio	R060A	AL80564A	
2× Gflex PCR Buffer (Mg2+, dNTP plus)	TaKaRa Bio	R060A	AL80564A	
ISOGEN	Nippon Gene	311-02501	75009K	
Sample Buffer Solution (2+Mercaptoethanol) (X4)	FUJIFILM Wako Pure Chemical Corporation	191-13272	WDP4995	
Pre-stained Protein Markers (Broad Range) for SDS-PAGE	Nacalai Tesque	02525	L9M9989	
5×Prime Script Master Mix	TaKaRa Bio	RR036A	AIE0440A	
Recombinant protein				
Ras·GTP (active Ras)-binding domain of human c-Raf	Dr. Kenji Tago (Jichi Medical University, Tochigi, Japan)	N/A	N/A	
Cell line				
FBD-102b cells (mouse cells)	Dr. Yasuhiro Tomo-oka (Tokyo University of Science, Chiba, Japan)	N/A	N/A	
Plasmids and vectors				
pcDNA3.1-N-EGFP as the N-terminal GFP-tag expression vector	GenScript	not described	N/A	
pXR001: EF1a-CasRx-2A-EGFP	Addgene	109049	N/A	
pSINmU6 as the oligonucleotide transcription vector	TaKaRa Bio	not described	N/A	
ACE2 intracellular domain sequences (5′ to 3′) inserted into the plasmid (pcDNA3.1-N-EGFP)				
Sense-KpnI-oligonucleotide for human ACE2 intracellular domain-KpnI 5′-GGTACCGGGATCAGAGATCGGAAGAAGAAAAATAAAGCAAGAAGTGGAGAAAATCCTTATGCCTCCATCGATATTAGCAAAGGAGAAAATAATCCAGGATTCCAAAACACTGATGATGTTCAGACCTCCTTTTAGGGTACC-3′ Antisense-KpnI-oligonucleotide for human ACE2 intracellular domain-KpnI 5′-GGTACCCTAAAAGGAGGTCTGAACATCATCAGTGTTTTGGAATCCTGGATTATTTTCTCCTTTGCTAATATCGATGGAGGCATAAGGATTTTCTCCACTTCTTGCTTTATTTTTCTTCTTCCGATCTCTGATCCCGGTACC-3′	This manuscript	N/A	N/A	
gRNA sequences (5′ to 3′) inserted into the plasmid (pSINmU6)				
Sense-BamHI-gLuciferase-ClaI gatccGCACCCGTGCAAAAATGCAGGGGTCTAAAACGGCGCCATTCTATCCTCTAGAGTTTTTTat Antisense-BamHI-gLuciferase-ClaI cgatAAAAAACTCTAGAGGATAGAATGGCGCCGTTTTAGACCCCTGCATTTTTGCACGGGTGCg	This manuscript	N/A	N/A	
Sense-BamHI-gACE2-93th-ClaI gatccGCACCCGTGCAAAAATGCAGGGGTCTAAAACGTCTTCAGCTTCCTGATTAAAGTTTTTTat Antisense-BamHI-gACE2-93th-ClaI cgatAAAAAACTTTAATCAGGAAGCTGAAGACGTTTTAGACCCCTGCATTTTTGCACGGGTGCg	This manuscript	N/A	N/A	
Sense-BamHI-gACE2-169th-ClaI gatccGCACCCGTGCAAAAATGCAGGGGTCTAAAACCACTCATCTTTTGGGCATTTTCTTTTTTat Antisense-BamHI-gACE2-169th-ClaI cgatAAAAAAGAAAATGCCCAAAAGATGAGTGGTTTTAGACCCCTGCATTTTTGCACGGGTGCg	This manuscript	N/A	N/A	
siRNA sequences (5′ to 3′)				
Sense chain for siLuciferase (siControl) GCCAUUCUAUCCUCUAGAG-dTdT Antisense chain for siLuciferase (siControl) CUCUAGAGGAUAGAAUGGC-dTdT	Yamauchi, J. et al. Exp. Cell Res. (2009) 315:2043-2052	N/A	N/A	
Sense chain for siACE2-128th GUUCACUUGCUUCUUGGAA-dTdT Antisense chain for siACE2-128th UUCCAAGAAGCAAGUGAAC-dTdT	This manuscript	N/A	N/A	
Sense chain for siACE2-169th GAAAAUGCCCAAAAGAUGA-dTdT Antisense chain for siACE2-169th UCAUCUUUUGGGCAUUUUC-dTdT	This manuscript	N/A	N/A	
RT-PCR primers (5′ to 3′)				
Sense primer for Arf1 (internal control) ATGGGTGGCTTTTTCTCAAGTATTTTTTC Antisense primer for Arf1 (internal control) TCACTGTCTGCTTTTCAGGGTTTC	This manuscript	N/A	N/A	
Sense primer for ACE2 ATGTCCAGCTCCTCCTGGTCCTTC Antisense primer for ACE2 TCAGCATAGAGTTTGCCCAGAATCCTTGAGTCATATG	This manuscript	N/A	N/A	

## Data Availability

Not applicable.

## Supplementary Materials

The following supporting information can be downloaded at: https://www.mdpi.com/article/10.3390/ncrna8030042/s1. Figure S1. siRNA and gRNA sequences.; Figure S2. ACE2 inhibition slightly promotes morphological differentiation in primary oligodendrocyte precursor cells.; Figure S3. ACE2 inhibition slightly promotes oligodendroglial cell morphological differentiation in FBD-102b cells.; Figure S4. The intracellular domain of ACE2 preferentially interacts with phosphatidylinositol-3 kinase α in a guanine-nucleotide-dependent manner.; Figure S5. The intracellular domain of ACE2 preferentially interacts with phosphatidylinositol-3 kinase β in a guanine-nucleotide-dependent manner.
