# Supplementary material for: CRISPR/CasRx-Mediated RNA Knockdown Reveals That ACE2 Is Involved in the Regulation of Oligodendroglial Cell Morphological Differentiation
Source: Noncoding RNA. 2022 Jun 6;8(3):42. doi: 10.3390/ncrna8030042 (PMC9229887; doi:10.3390/ncrna8030042)
Supplement: Supplementary file 1 [file ncrna-08-00042-s001.zip › Supple/Figure legends S1 to S5.pdf]

Figure legends S1 to S5

Figure S1. siRNA and gRNA sequences. (A) Target nucleotide number and its siRNA sequences and confirmation of knockdown efficiency by RT-PCR are shown. The siRNA for the 169th nucleotide sequence was used for experiments. (B) Target nucleotide number and its gRNA sequences confirmation of knockdown efficiency by RT-PCR are shown. The plasmid encoding gRNA for the 93rd nucleotide sequence was used for experiments.

Figure S2. ACE2 inhibition slightly promotes morphological differentiation in primary oligodendrocyte precursor cells. Primary oligodendrocyte precursor cells were treated with vehicle or MLN-4760 (1 nM) and were allowed to be differentiated for 0 or 3 days. The number of branches in cells is counted and shown (*, p<0.05; n=50 cells). Cells at 3 days following the induction of differentiation were collected, lysed, and immunoblotted with an antibody against PLP1, CNPase, SOX10, or actin. Band intensities were also compared to be depicted in graphs.

Figure S3. ACE2 inhibition slightly promotes oligodendroglial cell morphological differentiation in FBD-102b cells. FBD-102b cells were treated with vehicle or MLN-4760 (1 nM) and were allowed to be differentiated for 0 or 3 days. Differentiation efficiencies were divided into 3 categories and depicted in graphs (**, p<0.01; n=5 fields). The number of branches in cells is also counted and shown (**, p<0.01; n=50 cells). Cells at 3 days following the induction of differentiation were collected, lysed, and

immunoblotted with an antibody against PLP1, CNPase, SOX10, or actin. Band intensities were also compared to be depicted in graphs (**, p<0.01; n=3 blots).

Figure S4. The intracellular domain of ACE2 preferentially interacts with phosphatidylinositol-3 kinase α in a guanine-nucleotide-dependent manner. Cells were transfected with the plasmids encoding the GFP-tagged ACE2 intracellular domain and lysed. Cell lysates were immunoprecipitated with an anti-GFP antibody in the presence of 100 μM of GTP or GDP and immunoblotted with an anti-phosphatidylinositol-3 kinase α (PI3Kα) antibody. Immunoblots for total GFP-tagged proteins and PI3Kα proteins are also shown. Immunoreactive band intensities were depicted compared to the control in graphs (**, $p < 0.01$; n=3 blots).

Figure S5. The intracellular domain of ACE2 preferentially interacts with phosphatidylinositol-3 kinase β in a guanine-nucleotide-dependent manner. Cells were transfected with the plasmids encoding the GFP-tagged ACE2 intracellular domain and lysed. Cell lysates were immunoprecipitated with an anti-GFP antibody in the presence of 100 μM of GTP or GDP and immunoblotted with an anti-phosphatidylinositol-3 kinase β (PI3Kβ) antibody. Immunoblots for total GFP-tagged proteins and PI3Kβ proteins are also shown. Immunoreactive band intensities were depicted compared to the control in graphs (**, $p < 0.01$; n=3 blots).
