# Supplementary figures and images for: CRISPR/CasRx-Mediated RNA Knockdown Reveals That ACE2 Is Involved in the Regulation of Oligodendroglial Cell Morphological Differentiation
Source: Noncoding RNA. 2022 Jun 6;8(3):42. doi: 10.3390/ncrna8030042 (PMC9229887; doi:10.3390/ncrna8030042)

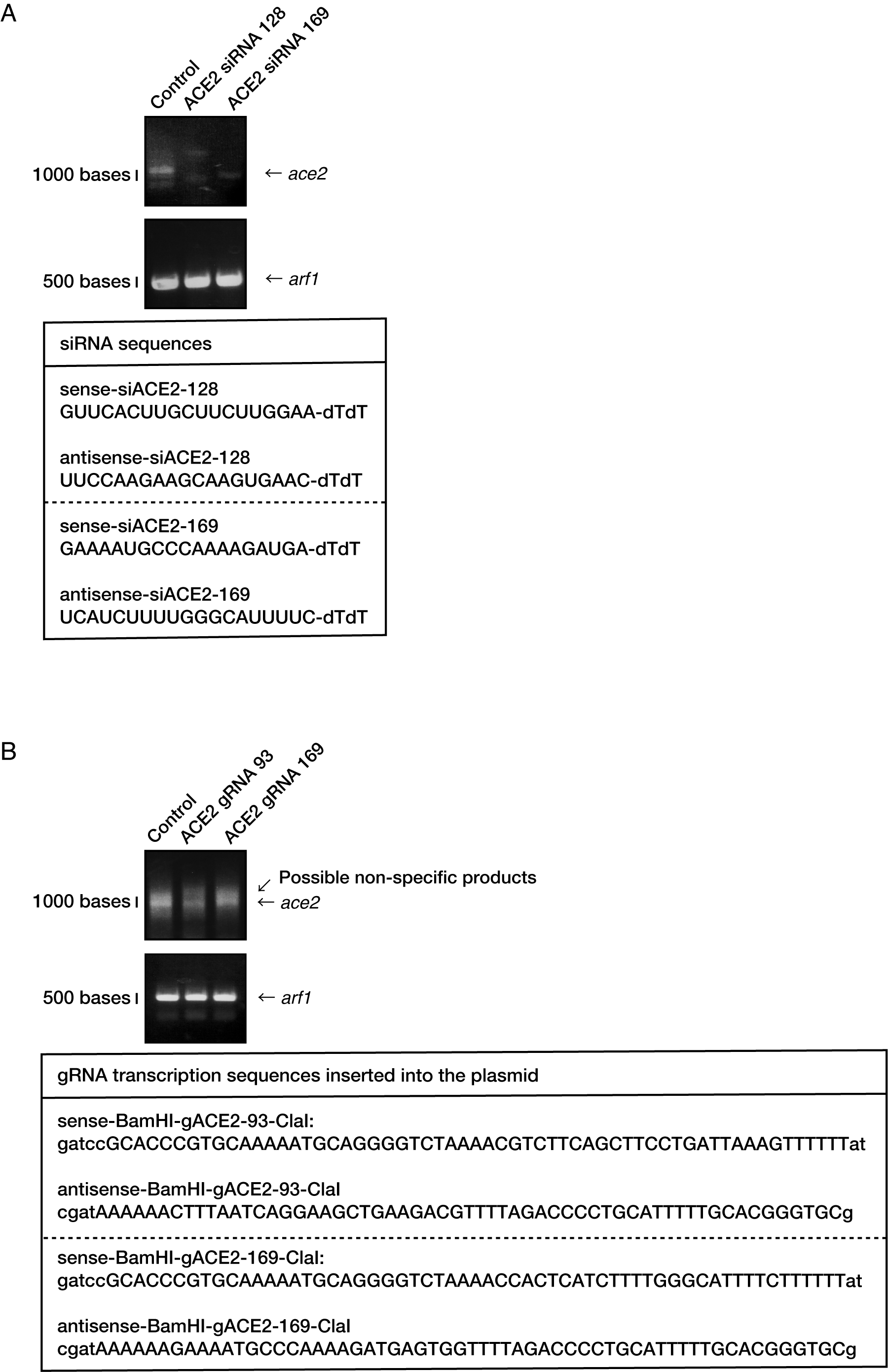

Supplement: Supplementary file 1 [file ncrna-08-00042-s001.zip › Supple/Figure S1.tif]

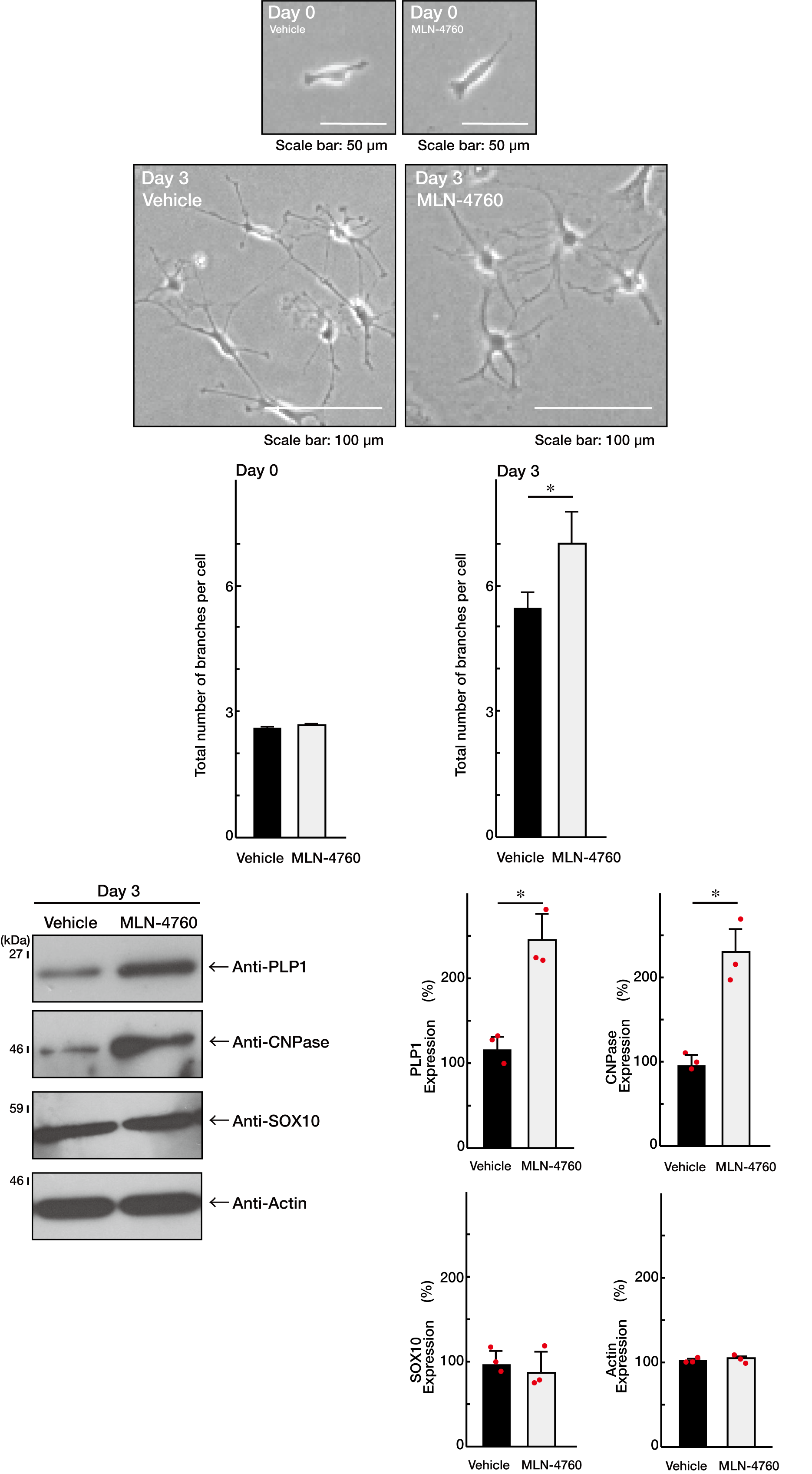

Supplement: Supplementary file 1 [file ncrna-08-00042-s001.zip › Supple/Figure S2.tif]

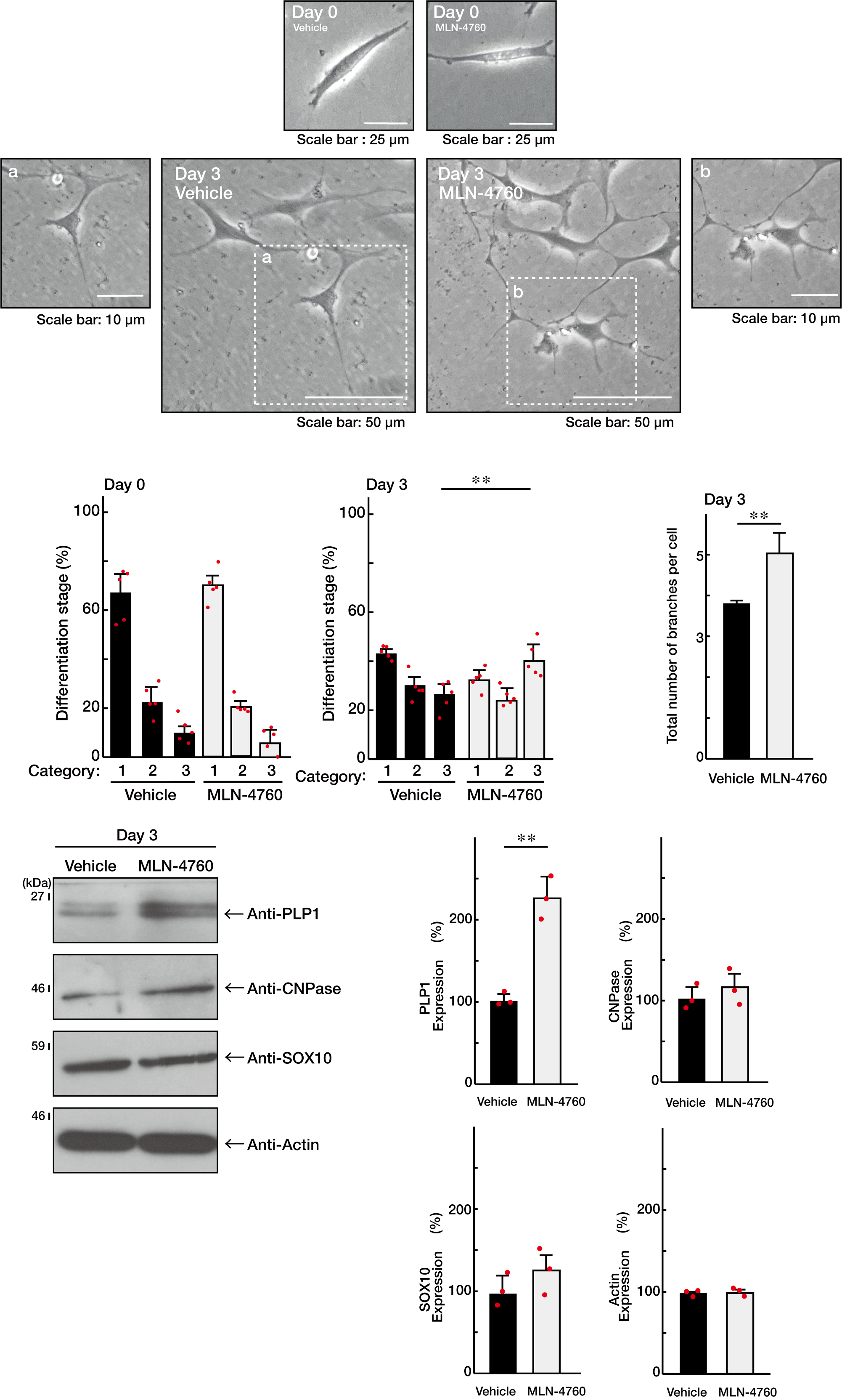

Supplement: Supplementary file 1 [file ncrna-08-00042-s001.zip › Supple/Figure S3.tif]

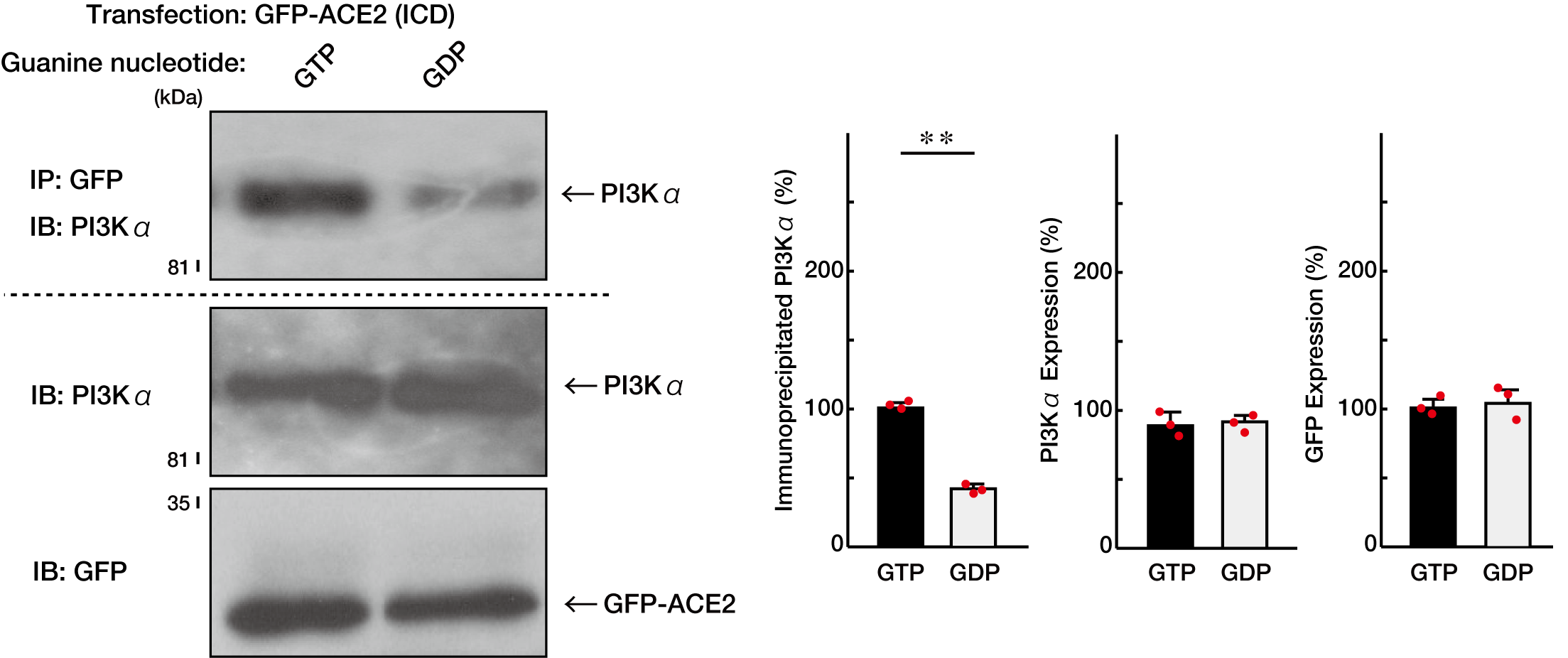

Supplement: Supplementary file 1 [file ncrna-08-00042-s001.zip › Supple/Figure S4.tif]

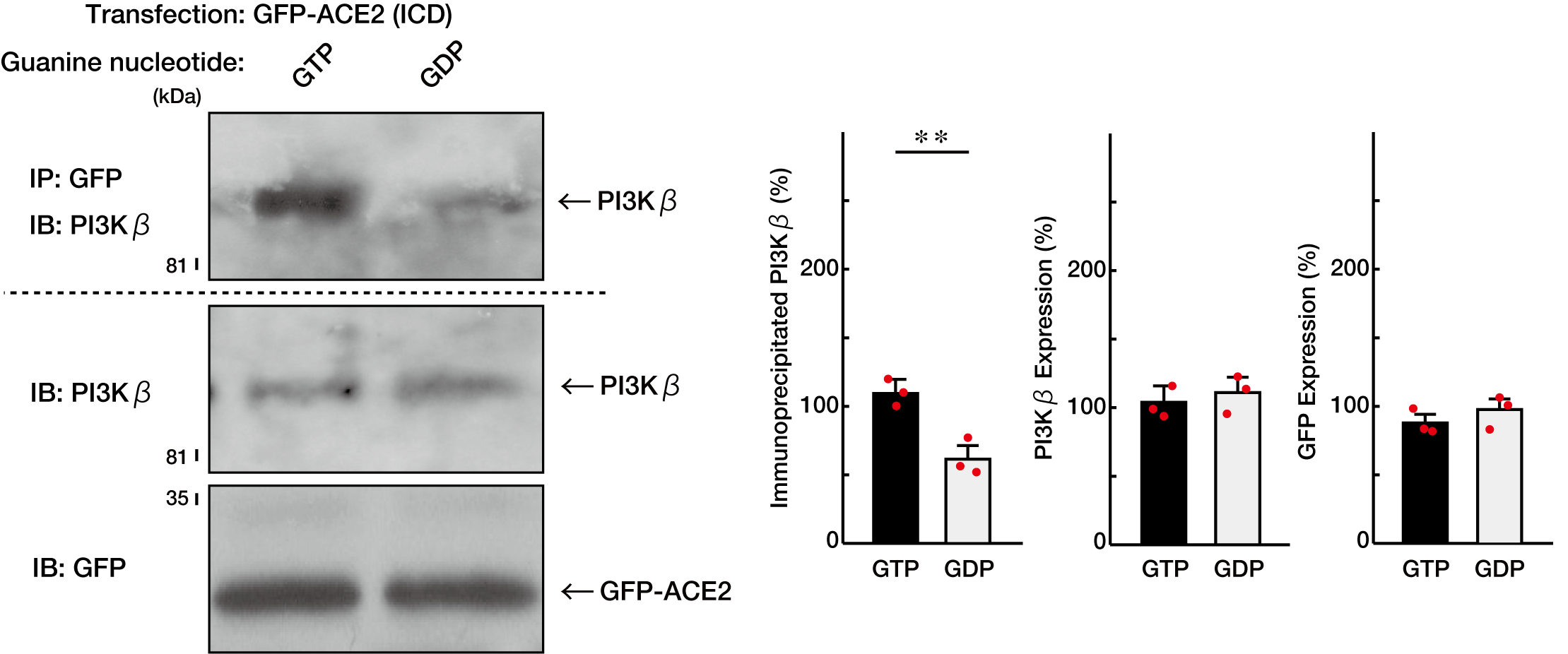

Supplement: Supplementary file 1 [file ncrna-08-00042-s001.zip › Supple/Figure S5.tif]
